# Growth, development, and health status of primary school students in Binzhou city: an analysis based on health examination data for 6-12-Year-olds

**DOI:** 10.3389/fpubh.2026.1757793

**Published:** 2026-03-24

**Authors:** Shuwei Li, Xuan Wang

**Affiliations:** 1 Department of Pediatrics, Binzhou People's Hospital, Binzhou, China; 2 Clinical Laboratory, Binzhou Medical University Hospital, Binzhou, China

**Keywords:** dental caries, epidemiology, obesity, overweight, primary school students, visual impairment

## Abstract

**Background:**

With socioeconomic development and lifestyle changes, the spectrum of health issues among Chinese children has shifted, with overweight/obesity and visual impairment emerging as major public health challenges. As a representative city in Northern Shandong Province, Binzhou requires localized data to understand the health status of its primary school students and to inform targeted intervention. This study systematically analyzes the growth, development, and major health problems among primary school students aged 6–12 years in Binzhou, aiming to provide a scientific basis for regional disease prevention and health promotion.

**Methods:**

A cross-sectional design was employed, drawing on health examination records from 4,281 primary school students aged 6–12 years in Binzhou. Data collected included age, sex, height, weight, vision, and dental caries. Statistical analyses were performed with SPSS. Categorical data were described as frequencies (percentages), and intergroup comparisons were assessed using the chi-squared test, with a significance level of α = 0.05.

**Results:**

A total of 4,281 primary school students were included, comprising 51.81% males and 48.19% females. The principal health problems identified were overweight, obesity, visual impairment, and dental caries. Issues related to stunted growth were minimal. The detection prevalences of overweight, obesity, color vision deficiency, and visual impairment differed significantly by gender (*P* < 0.05).

**Conclusion:**

The health issues among primary school students in Binzhou have shifted from traditional malnutrition toward lifestyle-related problems such as overweight, obesity, visual impairment, and dental caries. These conditions show high prevalence with distinct age- and gender-related patterns. Education and health authorities should implement precise, phased prevention and control strategies, with a particular emphasis on overweight, obesity, visual impairment, and dental caries in younger children.

Child health is the foundation of national health and a key determinant of a country's future development and overall population health. Primary school students occupy a critical period of growth and development, during which their health status is not only susceptible to multiple factors such as nutritional status, physical activity, learning environment, and behavioral habits but also serves as an important indicator of regional public health services and socioeconomic development. In recent years, rapid socioeconomic progress and substantial lifestyle changes in China have coincided with new and rising health challenges among children and adolescents. In particular, the prevalence of obesity and visual impairment has increased, highlighting these issues as pressing public health concerns (1–3).

According to the national health survey, the rates of overweight and obesity among Chinese school-age children continue to rise, and the prevalence of hypertension is trending toward younger ages. Moreover, the social stigma associated with childhood obesity often contributes to a range of psychological issues (4). Meanwhile, visual impairment remains prevalent with an earlier onset, adversely affecting both physical and mental health in adolescents (5). Oral health problems are also widespread among primary school students and negatively impact quality of life and academic performance (6).

As a major city in Shandong Province, China, Binzhou City represents a meaningful context for assessing child health in Northern Shandong. However, systematic municipal-level investigations with large samples and multiple health indicators remain scarce. Existing data are often fragmented across institutions and lack integration and in-depth analysis. Therefore, there is an urgent need for a comprehensive survey to establish localized baseline data.

This study adopts a cross-sectional design, analyzing health examination data from over 4,000 primary school students in Binzhou. It systematically characterizes the current status and distribution of growth metrics, vision, color vision, and dental caries, with comparisons across age groups and sexes. The findings aim to provide a scientific evidence to education and public health authorities for the development of targeted health promotion and intervention strategies, thereby contributing to the healthy development of children in this region.

## Methods

### Study population and data source

This study employed a retrospective cross-sectional design. Data were drawn from the routine health examination database for primary school students in the urban areas of Binzhou. It should be noted that the study sample was limited to students from primary schools in the urban areas of Binzhou, and did not include students from rural schools. This sampling framework was a design choice based on data accessibility and the completeness of the health examination system in urban areas. The inclusion criteria comprised all students aged 6–12 years with complete health examination records in the database. Prior to data analysis, we assessed the plausibility of height, weight, BMI, and visual acuity measurements. Exclusion criteria were: (1) records with missing key data, such as age, sex, height, and weight; and (2) records containing logical inconsistencies, such as implausible values for height, weight or visual acuity.

After screening, 4,281 eligible participants aged 6–12 years, covering grades 1–6, were included in the final analysis. All data were anonymized upon export, retaining only age, sex, and health examination indicators. The study protocol was approved by the Ethics Committee of Binzhou People's Hospital (YXKYLL-20250903), with a waiver of informed consent.

### Data content and definitions

The following health examination indicators were extracted and analyzed. ① Demographics: age and sex. ② Growth and development: height (cm) and weight (kg). Body mass index (BMI) was calculated as weight (kg) divided by height (m) squared (kg/m^2^). ③ Ophthalmology: uncorrected visual acuity for the left and right eyes, and color vision. ④ Oral health: presence of dental caries.

### Diagnostic and grouping criteria

Height and weight were measured using a calibrated mechanical stadiometer and electronic scale, with participants barefoot, no headwear, and wearing light clothing. Height was recorded to the nearest 0.1 cm, and weight to the nearest 0.1 kg. To ensure consistency and rigor, all raw examination values were re-evaluated according to current national standards during data analysis.

Nutritional status: Participants were classified into severe/moderate wasting, mild wasting, normal weight, overweight, and obesity groups based on age- and sex-specific BMI cut-offs from the Screening for Malnutrition among School-Age Children and Adolescents (WS/T 456-2014) and the Screening for Overweight and Obesity among School-Age Children and Adolescents (WS/T 586-2018).

Height: Stunting was defined using age-specific height cut-off values from the screening for malnutrition among school-age children and adolescents (WS/T 456-2014), classifying participants as stunted or normal.

Visual impairment: Visual acuity was assessed using a standard logarithmic visual acuity chart at a distance of 5 meters, with illumination meeting the required specifications. Defined as uncorrected visual acuity < 5.0 in either eye.

Color vision deficiency and dental caries were determined based on clinical diagnoses recorded in the health examination reports, and detection rates were calculated.

### Statistical analysis

All statistical analyses were performed using SPSS version 26.0. Categorical variables are presented as frequencies and percentages to describe detection rates. Group comparisons were conducted using the chi-squared test, with a significance level of α = 0.05.

## Results

A total of 4,281 valid samples aged 6–12 years were included in the final analysis. The cohort consisted of 2,218 males (51.81%) and 2,063 females (48.19%), yielding a male-to-female ratio of 1.07:1.

**Figure 1:**
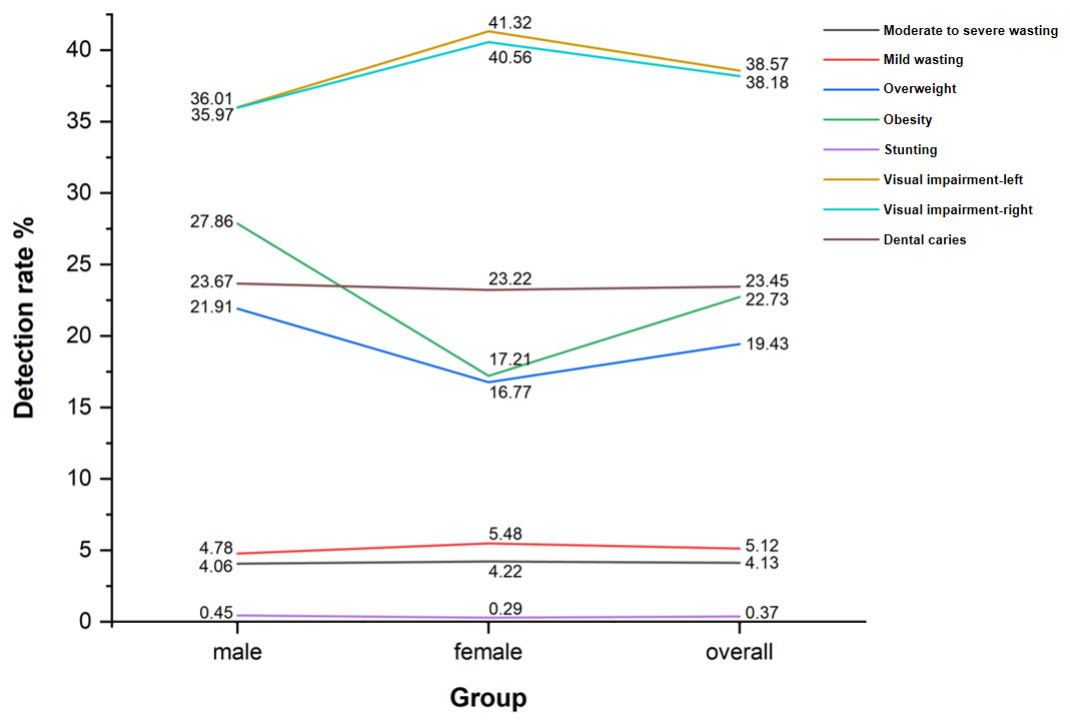
Differences in the detection rates of health problems by sex and population group.

Analysis of physical examination parameters identified overweight, obesity, visual impairment, and dental caries as the predominant health problems in this population. The detection rates of overweight, obesity, color vision deficiency, and visual impairment showed statistically significant differences between genders (Table 1, Figure 1).

**Table 1:** Differences in the distribution of different health problems by gender.

**Variable**	**Overall *N* = 4,281**	**Male *N* = 2,218**	**Female *N* = 2,063**	**χ^2^**	***P***
Moderate to severe wasting	177	90	87	0.067	0.795
Mild wasting	219	106	113	0.926	0.336
Overweight	832	486	346	18.18	*** < 0.001***
Obesity	973	618	355	69.10	*** < 0.001***
Stunting	16	10	6	0.252	0.615
Visual impairment-left	1,582	766	816	11.56	*** < 0.001***
Visual impairment-right	1,566	765	801	8.64	***0.003***
Color blindness	13	12	1	8.56	***0.003***
Color weakness	82	79	3	69.03	*** < 0.001***
Dental caries	1,004	525	479	0.116	0.733

Values in bold indicate a statistically significant difference (*p* < 0.05).

### Age-specific distribution of health problems

**Figure 2:**
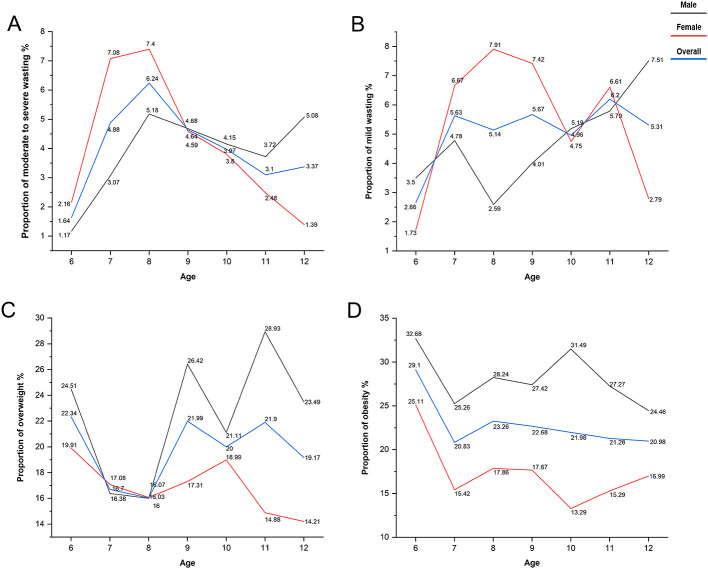
Variation in health problem detection rates across age groups. **(A)** Moderate to severe wasting; **(B)** mild wasting; **(C)** overweight; **(D)** obesity.

Regarding weight-related issues, the detection rates for moderate-to-severe wasting and mild wasting were consistently low across all age groups, peaking at 6.24%. In contrast, overweight and obesity were more prevalent, with overweight ranging from 16.03 to 22.34% and obesity from 20.83 to 29.10 across different ages (Figure 2).

**Figure 3:**
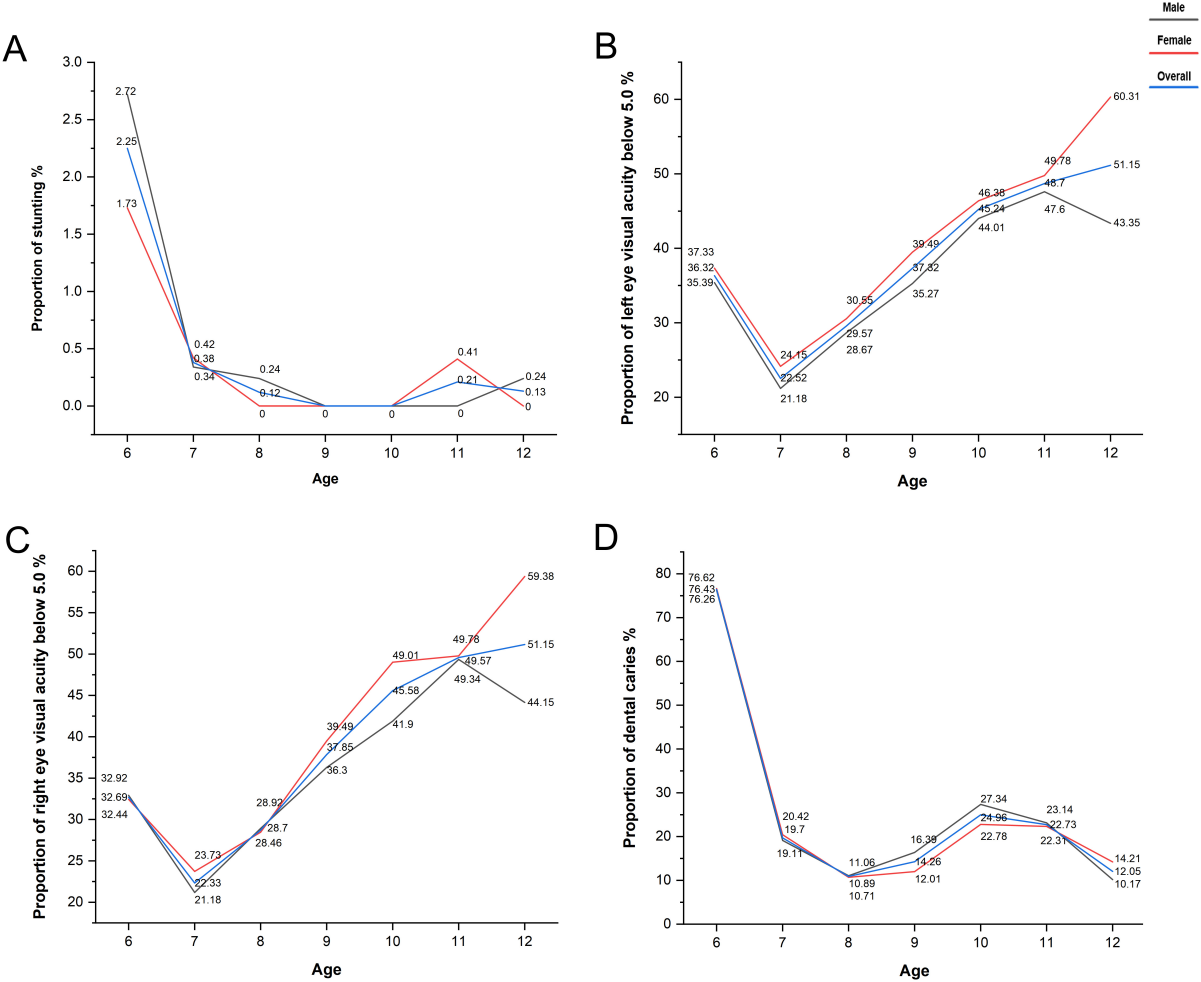
Variation in health problem detection rates across age groups. **(A)** Stunting; **(B)** visual impairment-left; **(C)** visual impairment-right; **(D)** dental caries.

Stunting was most prevalent in the 6-year-old group (2.25%), with rates remaining below 1% in children aged 7–12 years. The height of all children identified with stunting deviated from the standard reference value by less than 1.5 cm, suggesting a relatively modest overall impact (Figure 3).

For visual impairment, the detection rate for uncorrected visual acuity < 5.0 in either eye was notably high across all ages, starting at 22.52% and increasing with age to 51.15% by 12 years old (Figure 3).

The prevalence of dental caries was highest in the 6-year-old group at 76.43%, followed by a marked decline in subsequent ages, fluctuating between 10.89 and 24.96% (Figure 3).

## Discussion

This study systematically analyzed health examination data from 4,281 primary school students aged 6–12 years in Binzhou, revealing distinct patterns of growth, development, and health status among children in this region. With ongoing socioeconomic development and continuous improvements in public health services, traditional nutritional problems such as wasting and stunting have been effectively controlled, as evidenced by very low detection rates. These findings reflect substantial public health achievements in Binzhou in safeguarding children's basic nutrition and health. However, lifestyle-related health issues, such as overweight/obesity, visual impairment, and dental caries, have become increasingly prominent, displaying clear age and gender distribution patterns. From a public health perspective, this regional data, derived from a large sample, provide empirical evidence to inform local education authorities, health officials, and school health promotion programs. It indicates a need to reallocate resources toward preventing lifestyle-related chronic conditions, enhancing visual health surveillance, promoting oral health education, and supporting behavior change at the family level, while consolidating gains in traditional nutritional improvements.

Globally, rates of overweight and obesity are rising rapidly, with many countries experiencing substantial increases in childhood and adolescent obesity. If this trajectory continues, projections suggest that by 2050 nearly one-third of the world's children and adolescents could be affected by overweight or obesity. China, as the world's most populous country, faces a particularly severe situation, with estimates indicating over 600 million individuals affected by overweight (7, 8). In the present study, overweight and obesity prevalences were notably high in the surveyed population, substantially exceeding the national adolescent obesity rate of 7.9% reported by health authorities and aligning with concerns about the obesity epidemic among Chinese school-aged children (1). Moreover, numerous studies indicate that childhood and adolescent obesity often track into adulthood, with high BMI being a major risk factor for cardiovascular diseases, diabetes, and various cancers (9). As obesity rates rise, there is an associated increase in musculoskeletal injuries, such as falls, sprains, lower limb fractures, and joint dislocations (10). Meanwhile, children with obesity often experience ridicule due to their physique, which can trigger emotional disturbances. Such emotional problems may further exacerbate excess energy intake by affecting neuroendocrine regulation or leading to emotional eating, ultimately forming a vicious cycle of “obesity-emotional disturbance-worsening obesity” (11, 12). These findings collectively underscore the urgency of implementing targeted obesity prevention and control measures at regional levels.

The obesity epidemic is closely associated with rapid socioeconomic development, increased consumption of high-energy diets, and insufficient physical activity. In children and adolescents, obesity often reflects excessive dietary energy intake. In this study, detection rates of overweight and obesity were significantly higher in boys than in girls, a gender disparity consistent with prior reports (13). Potential underlying factors include differences in dietary patterns, greater sedentary time, and variations in the level and type of physical activity participation. These findings suggest that future obesity interventions should account for gender differences and provide more targeted health education and behavioral guidance for male students. Weight management for children with obesity should be guided by physicians or nutrition professionals (14).

Secondly, visual impairment was highly prevalent among children and showed a marked age-related increase, with detection rates exceeding 50% by age 12. This prevalence is substantially higher than in some earlier Chinese reports and indicates an earlier onset trend. Socioeconomic development, widespread use of electronic devices, and suboptimal visual behaviors contribute to the rising risk of refractive errors. Moreover, a markedly insufficient amount of outdoor activity time among school-aged children has been identified as a key environmental factor in myopia development and progression (15). This study also observed a higher detection rate of visual impairment among females compared to males, consistent with previous research and potentially related to inequalities in educational opportunities, access to health information, and utilization of ophthalmic services among females (16, 17). These results underscore a pressing need for rigorous visual health prevention and control measures among primary school students in Binzhou, including policies to ensure adequate outdoor activity time, strengthened home–school collaborative health management, and a focus on safeguarding girls' visual health.

The color vision assessment in this study revealed a notable gender disparity in the prevalence of color vision deficiency. The overall detection rates for color blindness and color weakness were 0.30 and 1.92%, respectively, which are lower than the national average. Importantly, detection rates were significantly higher in males (color blindness: 0.54%; color weakness: 3.57%) than in females (color blindness: 0.05%; color weakness: 0.15%). This distribution pattern is consistent with the established X-linked inheritance of color vision deficiency (18, 19).

Although color vision deficiency is not generally considered a severe medical condition, it can impact children's academic performance, daily activities, and future career choices (20). Therefore, early screening and identification are of great importance. For identified students, targeted school accommodation and psychological support should be provided, and genetic counseling services should be offered to their families. Additionally, guidance on educational and career planning should be provided to mitigate the potential impact of color vision deficiency on individual development and social participation.

Dental caries is the most common chronic non-communicable oral disease in childhood and is one of the key non-communicable diseases targeted for global control by the World Health Organization (6). In this study, the detection rate of dental caries remained relatively high, peaking in the 6-year-old age group and then gradually declining with increasing age. This age-related pattern is consistent with the findings of the National Student Physical Fitness and Health Surveillance. The main reasons for this phenomenon include inadequate oral health awareness in early childhood, high-sugar dietary patterns, and limited parental recognition of the harms of deciduous caries. In addition, during the mixed dentition period, the gradual exfoliation of deciduous teeth and the eruption of permanent teeth contribute to a relatively lower risk of caries in newly erupted permanent teeth (21, 22). At the same time, as children grow older, oral hygiene habits become more established, self-care awareness improves, and parents place greater emphasis on oral health. Although the detection rate of dental caries generally declines with age and has fallen below the average observed in some other countries, it remains at a non-negligible level among upper-grade primary school students in Binzhou (23, 24). This increases the healthcare burden while also negatively impacting children's physical and mental wellbeing. These findings underscore the need for ongoing, systematic, and age-specific oral health interventions throughout the entire primary school period.

Based on these findings, we propose the following recommendations. First, education and health departments should collaborate to place obesity prevention and visual impairment control at the core of school health programs, developing and implementing targeted, gender- and age-specific health promotion strategies (14). Second, early intervention should be strengthened, with particular emphasis on fostering oral hygiene and proper eye-care habits during the early years of primary school. The results provide localized scientific evidence to inform precise child health policy in Binzhou and similar regions. Future research should further explore behavioral and environmental determinants underlying these health issues to guide more effective interventions.

### Limitations

This study has several limitations. First, as a cross-sectional survey, it can identify associations between health outcomes and factors such as age and gender but cannot establish causality. Second, the data were primarily derived from routine health examinations and did not include direct assessments of behavioral risk factors, such as dietary patterns, physical activity, sleep duration, and visual hygiene behaviors. This limitation constrains our ability to elucidate the mechanisms linking these health problems to modifiable behaviors. Third, the study sample was drawn exclusively from urban schools in Binzhou, with no rural students included. Consequently, the findings reflect only the health status of urban children and cannot be generalized to the entire primary school population across the city. Considering potential urban-rural differences in growth and development, lifestyle behaviors, and access to health services, future studies should include samples from both urban and rural areas to enable comparative analyses. Fourth, the assessment framework of this study primarily focused on indicators of physical health, failing to concurrently examine the increasingly prominent mental health issues among children. Existing research demonstrates that mental health is an indispensable component of overall health, and the absence of such data limits the capacity of this study to provide a more comprehensive evaluation of children's health status.

To address these limitations, future research should expand the sample size and include comparisons between urban and rural areas. In addition to health examinations, integrate multiple data collection methods (e.g., questionnaires, objective activity monitoring) and conduct long-term longitudinal follow-up to characterize the dynamic trajectories and determinants of children's health issues, while also assessing children's mental health status. Furthermore, multi-factor models incorporating environmental exposures, lifestyle factors, and genetic background could be developed to more precisely evaluate the effectiveness of interventions and to explore the underlying biological and psychosocial mechanisms.

## Conclusion

This study demonstrates that the primary health threats to primary school students in Binzhou have shifted toward lifestyle-related issues, including overweight, obesity, visual impairment, and dental caries. These health problems exhibit high prevalence and distinct age- and gender-specific distribution patterns. Consequently, traditional, generalized health promotion models are no longer sufficient. There is an urgent need for multi-sectoral collaboration among education, health, and related sectors to develop and implement precise, phased, and targeted prevention and control strategies. Specifically, overweight, obesity, and visual impairment prevention and control should be central to school health initiatives, complemented by strengthened oral health interventions in the lower grades of primary school. The findings provide crucial scientific evidence to inform child health policies in Binzhou and in regions with similar socioeconomic contexts.

## Data Availability

The raw data supporting the conclusions of this article will be made available by the authors, without undue reservation.
